# Supplementary material for: Smooth Interpolating Curves with Local Control and Monotone Alternating Curvature
Source: Comput Graph Forum. 2022 Oct 6;41(5):25–38. doi: 10.1111/cgf.14600 (PMC9827861; doi:10.1111/cgf.14600)
Supplement: Supplementary file 1 — Supplement Material [file CGF-41-25-s001.zip › Local-Smooth-Interpolating-MonoCurvature/extern/clothoids/docs/api-matlab/program_listing_file_TriTriOverlap.m.html]

Program Listing for File TriTriOverlap.m — Clothoids v2.0.9

### Navigation

- index
- toc
- Clothoids »
- Program Listing for File TriTriOverlap.m

# Program Listing for File TriTriOverlap.m¶

↰ Return to documentation for file (`TriTriOverlap.m`)

```
%> TriTriOverlap:  Check if two triangles overlap
%>
%> **Usage:**
%>
%>     intersect = TriTriOverlap( p0, p1, p2, q0, q1, q2 );
%>
%> **On input:**
%>
%> - p0, p1, p2 = coordinates of first triangle
%> - q0, q1, q2 = coordinates of second triangle
%>
%> **On output:**
%>
%> - intersect = true if the triangle overlap
%>
%======================================================================%
%                                                                      %
%  Autor: Enrico Bertolazzi                                            %
%         Department of Industrial Engineering                         %
%         University of Trento                                         %
%         enrico.bertolazzi@unitn.it                                   %
%                                                                      %
%======================================================================%
function intersect = TriTriOverlap( p0, p1, p2, q0, q1, q2 )
  error('this function is mex only. Run CompileClothoidsLib.m script to build')
end
```

### Quick search

### Table of Contents

- Matlab Interface Manual
- C++ API
- MATLAB API

«
hide menu

menu
sidebar
»

### Navigation

- index
- toc
- Clothoids »
- Program Listing for File TriTriOverlap.m

© Copyright 2021, Enrico Bertolazzi and Marco Frego.
Created using Sphinx 4.2.0.
